# Automation of a plane grating spectrograph

**DOI:** 10.1155/S1463924696000028

**Published:** 1996

**Authors:** Carlos Roberto Bellato, Jarbas J. R. Rohwedder, Ivo M. Raimundo, Jr., Celio Pasquini

**Affiliations:** Instituto de Química Universidade Estadual de Campinas Caixa Postal 6154 Campinas SP CEP 13083 970 Brazil


Journal of Automatic Chemistry, Vol. 18, No. 1 (January–February 1996), pp. 7–15

### Automation of a plane grating spectrograph

**Carlos Roberto Bellato, Jarbas J. R. Rohwedder, Ivo M. Raimundo Jr. and Celio Pasquini***

*Instituto de Química, Universidade Estadual de Campinas, Caixa Postal 6154, CEP 13083 970, Campinas, SP, Brazil*

*A Zeiss model PGS-2 plane grating spectrograph was automated by replacing its photograph film detection system with an EGG-Reticon 1024S photodiode array (PDA) and by controlling the main instrument functions through the use of a home-made interface connected to a microcomputer. The array was cooled by four Peltier elements to allow integration of the emitted light for up to 40 s. The interface performs data acquisition from the sensor array, controls the grating position and the excitation source. A QuickBasic 4.5 program manages the interface, for data storage and treatment, and allows a graphical display and user-friendly interaction. Results show an absolute standard deviation for the wavelength localization of ±0·036 nm, and a spectral resolution of 0·05 nm at 443 nm when a 651 grooves/mm grating is employed. In each scan, the sensor array can collect data in a 18·7 nm wide window. Sensitivity was inferred from experimental data that showed an accurate localization of emission lines for Sn and Cu present in metal alloys at 0·006 and 0·03%, respectively. Quantitative results obtained for determination of Mn in steel samples and Pb in aqueous solution are also presented.*

### Introduction

Plane grating spectrographs are very robust optical instruments which have been used in analytical spectrophotometry for more than 40 years [1, 2]. Spectrographs measure the wavelength and intensity of the atomic emission of elements under an electrical excitation arc or spark source, which provides accurate qualitative information directly from solid samples such as metal alloys. The intensity of the light emitted at characteristic wavelengths can be used to find out the concentration of the element in the sample.

Early instruments used the human eye as the detector [2]. Photograph film became popular during the 1960s and 1970s and it is still used today to record the emission spectrum, including the intensities of the atomic lines [3]. Photographic films, especially developed for spectrographs, show very good sensitivity and resolution [4] but they are cumbersome and rely on the capabilities of the analyst. Also, if a quantitative determination is needed, an optical densitometer is required.

Despite the quality of the information that a film-based detector spectrograph can offer, its popularity declined when modern instruments, such as Inductively Coupled Plasma (ICP), became available along with new automatic, computer controlled, data acquisition and treatment systems. However, many research and routine laboratories still have spectrographs, although some of them have been deactivated. Nobody, however, could deny the usefulness of the information that could be given by these instruments to help in the analysis of a number of samples. Although it is not a perfect instrument for quantitative analysis, mainly due to the lack of reproducibility of its excitation source (minimized by the use of internal standards), the simple survey information it provides on relative concentrations of elements could help in the development of more accurate quantitative procedures using, for example, an ICP or a graphite furnace atomic absorption instrument.

The first move towards automation of the quantitative spectrographic procedure was achieved by digitizing the analogue output of optical densitometers used to find out the intensities of the atomic lines printed on the photographic film [5].

With the advent of the modern array of sensors, mainly the photodiode arrays (PDA), some authors recognized that the old film-based detection spectrograph could be modified to replace their detection systems [6–10]. More recently, Charge Coupled Devices (CCD) and Charge Injection Devices (CID) are being used with spectrographs [11, 12], although these are expensive and their size is not suitable for direct use with the optics of old fashioned spectrographs.

Some studies have involved only one spectral window [6–9], while others replaced the photograph film to observe a very wide wavelength range using up to nine PDAs [9, 10]. Clearly a number of people believe that it is worth making some effort to modernize old spectrographs because, with computer control and the new sensors on the market, they can still have a place in a modern analytical laboratory. To support this vision are features as the multi-elemental capability of such modified (modernized) instruments, their ease of use with raw solid samples (mainly metal alloys), the possibility of replacing the old arc/spark excitation sources with modern ICP sources while keeping the usually excellent optics of the spectrographs and, finally, the possibility of complete automation which would allow the spectral information to be used in modern data treatment software as expert systems [10, 13], pattern recognition [5, 14] and multivariate calibration [15, 16].

This paper describes the automation of a plane grating Zeiss PGS-2 spectrograph; the main feature is the replacement of the photograph film with a 1024 photodiode array sensor. A high degree of automation of the spectrograph has been achieved at a relatively low cost.

### Experimental

The Zeiss PGS-2 spectrograph in its most usual configuration employs a grating containing 651 grooves/mm and has a 2·075 m optical path which projects the dispersed entrace slit image in a 24 cm wide output aperture. Originally, a glass supported photograph film was placed at this plane to record the emission lines of elements

* *Correspondence to Celio Pasquini.*

0142-0453/96 $12.00 © 1996 Taylor & Francis Ltd.

present in samples. The excitation source is supplied by a power module and a d.c./a.c. arc or spark are available. The first stage of the present work was to identify the functions that could be automated without needing extensive modifications to the hardware of the original instrument.

To keep the costs of the proposed automation down, it was decided to use only one RL-1024S sensor whose photodiodes spread in a 2·54 cm long row. The automation should allow the grating to move, under computer control, to give access to a wider wavelength range when necessary. Another important function to be automated is the control over the power supplier so that the sample could be excited for a user selected time interval. Unfortunately, the selection of the kind of excitation (arc or spark) and other parameters associated with the excitation source, such as the intensity of current, pulse frequency, capacitance and inductance, are all commanded, in the original instrument, by heavy manual dials which were not automated at this stage.

*The automated spectrograph*

Figure 1 shows an overall view of the spectrograph after automation. An IBM-386 compatible microcomputer supplied with a math coprocessor, 340 Mb Winchester, 8 Mb RAM, was used. A home-made parallel user port and an addressable interface were employed to perform the analogue-to-digital data domain conversion and to act on electromechanical devices employed to control the spectrograph [17, 21].

*Computer interface*

The computer interface employed in the automation of the spectrograph was used to externally address the devices necessary to control and to perform data acquisition from the PDA sensor. Figure 2 shows the hardware used both for data acquisition and to control the power supplier of the excitation source. A detailed explanation of how

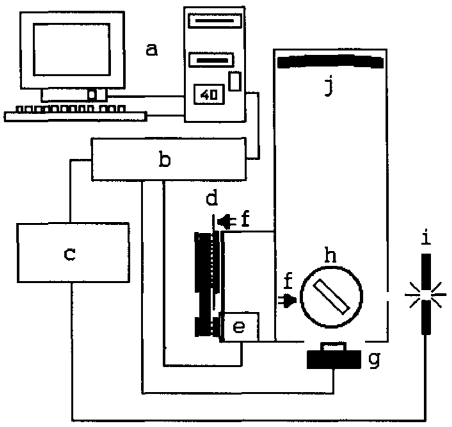

*Figure 1. Overview of the automated spectrograph. Where a: IBM-386 compatible microcomputer; b: controller and data acquisition interface; c: excitation power supplier; d: compact disk; e: stepper motor for grating positioning; f: reflexive optical switches; g: photodiode array sensor; h: grating support plate; i: excitation source; and j: spectrograph mirror.*

this kind of interface works can be found in references 19 and 21. The interface has a 12 bit analogue-to-digital converter (AD 7672KN-3) supplied by a very good reference voltage source (AD 588).

*Photodiode array sensor*

A photodiode array (PDA) sensor (EGG-Reticon RL-1024S) was used to record the intensity and wavelength of atomic emission lines. A main control board (RC 1000) and a satellite board (RC 1001), that contain the socket for the PDA, were also employed. The array of sensors was positioned at the middle of the output aperture of the spectrograph.

The main board provides the control signals to extract the 1024 analogue signals from the PDA output as a voltage peak through a sample-and-hold circuit. The board provides ways to synchronize the analogue output with an external A/D converter. However, the internal clock was bypassed and the control signals were started by setting the on board deep switches [22]. These signals, used to start a sequential scan of the 1024 sensors and to time the reading sequence, were generated, under computer software control, through the home-made interface.

The PDA and its companion boards can couple with scan rates as fast as 100 kHz. However, it was anticipated that long integration time intervals would be necessary to obtain suitable signal intensities in the spectrograph. Therefore, the control of the clock signal, although slower when made by the interface, is suitable for the application. The maximum scan frequency obtained by the present system is 2 kHz.

As long integration time intervals were predicted, the sensor array had to be cooled using Peltier elements placed on the back of the PDA's integrated circuit. The cooling devices were adapted using an aperture present in the satellite board that holds the array. Four 10 $mm^2$ Peltier elements were employed.

The sensor was cooled to about 10°C in a room kept at 25°C. The effect of the cooling system was to reduce the dark signal for a 15 s integration time from an average 2700 to 1300 units of the 12 bits A/D reading. Integration time, as long as 40 s can be used as the average dark signal reaches values around 3000 A/D units. In addition, the noise associated with each sensor readout (expressed as the absolute standard deviation evaluated for 10 readings for 20 selected diodes) decreases from an average $\pm 18$ to $\pm 6$ A/D units when the array is cooled.

*Grating position control*

Automatic control of the plane grating movement was effected to extend the wavelength range that the PDA can observe. As many spectrophotometers use the same manual approach to wavelength selection through angular movement of the dispersing element, this adaptation should be useful for other applications (see later).

The PGS-2 spectrograph controls its grating position through a dial that moves a threaded axis and this, in turn, moves a circular plate containing the grating mounting. In order to control the grating position automatically, two reflexive optical switches (RS 307-913) were adapted in the spectrograph. One of them follows the movement of the dial and the other was placed near the internal plate holding the grating (see figure 1). A stepper motor (3·5°/step) was employed to move the dial through a toothed belt wrapped around a gear fixed directly in the dial. A compact disk ($3\frac{1}{2}$ inch diameter) was placed between the gear and the dial with its unlabelled side facing the reflexive opto-switch. A fine radial trace was scratched onto the surface of the disk to act as a mark for the controller. The gear at the stepper motor and the gear dial was a 1:10 diameter ratio.

To locate the starting position for a wavelength scan, the software first moves the stepper motors backwards and looks at the internal optical switch to find the mark in the grating supporting plate. Then the stepper motor is moved forward and the external optical switch flags the mark in the compact disk. Using the two optical switches, the system can always find the starting position of the grating and shade the 200 nm wavelength region on the array surface. After being in the start position, the number of steps sent to the motor is used to move the grating to any user-selected wavelength window. Changes in the direction of movement of the motor do not produce a reproducible positioning due to both the accumulative error caused by the missing steps and to the mechanical faults that can occur in a treaded moving system. So each time a scan procedure is finished and each time the system is turned on, the grating is returned to its starting position.

### *Control of the excitation source*

The excitation sources as on/off function and other mechanical devices are controlled in the PGS-2 by push-buttons. The excitation source can be also controlled by a wire remote control. To transfer the on/off control to the computer, electromechanical switches have been added in the interface and connected to the remote control of the instrument. The electromechanical switches were controlled by logical TTL level signals generated under software control.

### *Wavelength calibration*

As only one diode array was employed as sensor, the instrument can only scan a wavelength window that is about 18·7 nm wide when the standard grating (651 grooves/mm) is employed. The grating can be moved under computer control to access the wavelength range from 200 to 640 nm; this movement is made ensuring that there is always a superposition of at least 1·8 nm in order not to lose any information. Four hundred and twenty nine steps were necessary to move from one to the next window. About 26 windows must be read to scan the whole range. At first it was assumed that only three equations could be used for the wavelength identification:

$$W_{1,j} = (j \times 1024 \times D) - S + W_{1,1} \qquad j \geqslant 2 \tag{1}$$

$$W_{1024,j} = W_{1,j} + (1023 \times D) \tag{2}$$

$$W_{N,j} = W_{1,j} + ((N - 1) \times D) \qquad 1 \geqslant N \leqslant 1024 \tag{3}$$

where $W_{1,j}$ is the initial wavelength over the first diode in the $j$ window, $D$ is the average reciprocal dispersion expressed in nm/diode, $S$ is the average wavelength superposition from one window to the next, and $W_{1,1}$ is the initial wavelength over the first diode in the first window. $W_{1024,j}$ is the wavelength over the last diode in the $j$ window. $W_{N,j}$ is the wavelength over the $N$th diode of the $j$ window. $W_{1,1}$ was estimated using two well-known emission lines of Cu that occur in the first window. The values of $D$ and $S$ were found by using the average of 10 values obtained for 10 windows calculated by looking at two well-defined emission lines in those windows.

However, mechanical differences along the thread of the axis that moves the grating supporting plate, together with the slight non-uniformity of the reciprocal linear dispersion over the entire wavelength range, meant that a more accurate procedure involving calibration of each window was necessary. Therefore, equations 1, 2 and 3 were used only to guide the initial positioning in a given window and to help in the selection of the element to be used in the calibration of that window. When positioned in a window, an element or a mixture of elements known to have at least two spaced and intense emission lines ($W_1$ and $W_2$), in the range encompassed by that window were excited by a d.c. arc and the intensity pattern of the 1024 sensors was acquired. The diodes ($n_1$ and $n_2$) over the peak of the emission lines were observed and were taken for the calibration. The initial wavelengths, $W_{1,j}$ and the reciprocal dispersion $D_j$ for that window was found by:

$$D_j = \frac{(W_2 - W_1)}{(n_2 - n_1)} \tag{4}$$

$$W_{1,j} = W_1 - (n_1 \times D_j) \tag{5}$$

When more than two well-defined emission lines were found in a window, they were included in the calibration procedure. After calibration, any wavelength in the range of that window can be obtained by the equation:

$$W_{N,j} = W_{1,j} + ((N - 1) \times D_j) \qquad 1 \leqslant N \leqslant 1024 \tag{6}$$

After total calibration, 26 values for $W_{1,j}$ and $D_j$ were stored in a file; they are used to convert the diode number to wavelength for each window that the user selects for a scan.

### *Software resources*

A program in Microsoft QuickBasic version 4.5 performs data acquisition, data treatment and controls the grating position and the excitation source. The user can select how many spectral windows are to be scanned in the data acquisition procedure and the user defines which windows, from the 26 available, to scan. The pre-burning and the integration time intervals are also set by the user. An optional dark signals subtraction function can be employed.

Spectral data for each window are shown in graphic form on the computer screen. Page up/down keyboard control can be used to display the windows sequentially. The user can alter the scale to zoom in on the spectral data. Data comparison can be performed by superimposing up to 10 windows obtained in distinct data acquisition procedures.

A graphic cursor can be moved across the spectra [17], while the wavelength, the diode number and the intensity, related with the cursor position are shown on the screen.

The main subprogram of the software package is the data acquisition procedure shown in appendix 1. To perform the 1024 readings from the PDA, the subprogram first perform a false very fast scan to reset all the sensors (SUB zero) and starts to count the integration time interval. Then the array is scanned again (SUB read-array) and the intensities are stored in an indexed variable and also saved in user named files.

For quantitative purposes, the user can select to average up to 10 spectra (obtained in the same window to improve the signal-to-noise ratio) and two peaks can be selected (one for the analyte and other for an internal reference). The analytical signal can be used as the maximum intensity or as the integral under the peak. In this last option, the diodes to be integrated are selected by using the graphic cursor.

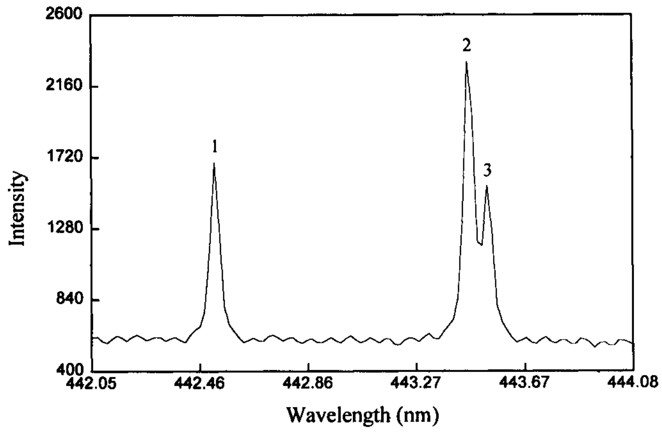

*Figure 3. Enlarged portion of an emission spectrum for Ca showing the resolution obtained in the automated spectrograph. (1) 442·544 nm; (2) 443·496 nm and (3) 443·569 nm respectively were found over the diodes 254, 306 and 310.*

### Results and discussion

The goal of spectrograph automation was to keep costs as low as possible, while preserving the qualitative and quantitative capabilities of the instrument. The automated instrument was initially evaluated for its reproducibility in terms of measured wavelength, and for overall resolution when a 651 grooves/mm grating is employed. The reproducibility and sensitivity achieved by the new sensor were investigated.

### *Evaluation of the instrument for use in qualitative analysis*

Qualitative use of the spectrograph is essentially based on accurate and reproducible wavelength localization and on its spectral resolution. The overall spectral resolution of the instrument was found by looking at pairs of emission lines whose wavelength distances were of the order of the ideal resolution as predicted by the ratio between the width of one photodiode (25 μm) and the linear reciprocal dispersion (0·730 nm/mm) expected on the basis of the instrument optics under standard conditions (2·075 m optical path, 651 grooves/mm grating). This ratio is equal to 0·018 nm per diode. Of course, the actual resolution should be worse than this. Figure 3 shows an enlarged view of an emission spectrum of Ca. The spectrum contains two emission lines (at 443·496 and 443·569) that can be used to access the actual spectral resolution of the instrument. The final conclusion regarding the spectral resolution is that, for the best case of intense signals, resolution can reach 0·05 nm. These figures could be improved by using instrument resources that improve the linear reciprocal dispersion as a grating with more grooves per mm, by double passing the light beam and/or by using higher orders.

The precision on wavelength localization was found by looking at some characteristics and well resolved lines of elements that appear in 10 windows spread over the entire spectral range achievable by the instrument. Ten measurements of the wavelength of the maximum of emission lines were made to estimate the standard deviation of their position. The grating, for each measurement, was moved to the starting point and then the suitable number of steps were sent to the motor to reach the desired window. Five points were collected this way. The other five were obtained by moving to the desired window passing through and stopping at any preceding window, simulating a wide range multiple window scan. The calibration equations were used to find out the wavelengths of the maximum emission intensities. The average standard deviation observed was of $\pm 0{\cdot}036$ nm, meaning that the mechanical movement of the grating can reproduce the wavelength position within $\pm 2$ photodiodes error.

Figure 4 shows spectra obtained for two metal alloys and a clay sample excited under a d.c. arc at 10 A for 15 s in the automated spectrograph. These data can be used to infer on the sensitivity of the automated instrument by, for example, noting that it is possible to locate characteristic emission lines for Sn and Cu present in a steel sample at concentrations as low as 0·006% and 0·03%, respectively. The spectrum in figure 4(*c*) demonstrate the utility of the spectrograph in a very fast survey on the elements present in a clay sample, pointing out (based on the relative intensities of the emission lines) the major and minor components of the sample.

### *Evaluation of the instrument for use in quantitative analysis*

In the automated spectrograph, the intensities of the emission lines are quickly available and can be treated to perform a quantitative evaluation of the emitting specimens. The usual procedure requires the use of an internal standard emission line to correct both for poor stability of the excitation source and sample excitation conditions (as distance between electrodes) [1]. The relative poor reproducibility achieved by the arc/spark excitation sources, when compared with modern sources like ICP, are compensated for in that solid samples are used directly without the need for any pre-treatment or potentially contaminating reagents. Also, the fact that a relative large window (18·7 nm) is observed each time, helps in the location of a suitable reference line emitted

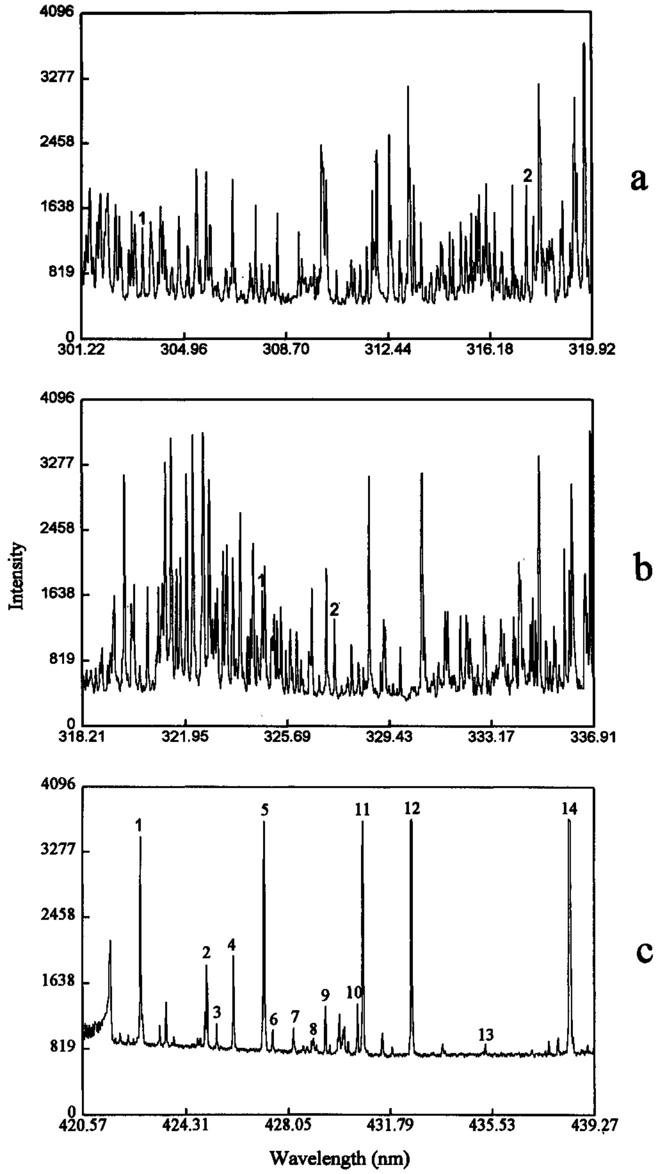

*Figure 4. Spectral windows for metallic alloys (a and b) (Bureau of Analyzed Samples Ltd) and a clay sample (c) showing the qualitative performance of the automated spectrograph. a: (1)—303·41 nm; (2)—317·50 nm characteristic emission for Sn present in the sample SS466 at 0·006%. b: (1)—324·75 nm characteristic emissions for Cu present in the steel sample SS465 at 003%. c: (1)—442·67 for Ca; (2)—425·08, (4)—426·05, (5)—427·18, (7)—428·24, (9)—429·41, (11)—430·79, (12)—432·58, (13)—435·27 and (14)—438·36 nm for Fe; (3)—425·44, (6)—427·48, (8)—428·97 nm for Cr; (10)—430·59 for Ti, all elements identified in one scan in a clay sample.*

by elements already present in the sample or added in the sample solution.

The automated spectrograph was evaluated for its quantitative performance in the determination of Mn directly in killed steel samples and Pb in aqueous solution by using the rotating disc technique [23–25].

Six test samples of killed steel containing Mn in the range 0·2–0·9% were submitted to an a.c. spark excitation (0 μH, 40 μF, 2 Ω and 100 Hz), employing an entrance slit of

*Table 1. Experimental results obtained for Mn determination in killed steel samples. The area ratios were calculated by using the integrated intensities centered at 293·31 nm (diodes 517 to 526) for Mn and 292·66 nm (diodes 476 to 493) for Fe.*

| Mn (%, w/w) | Integrated intensity for Mn, arbitrary units | Integrated intensity for Fe, arbitrary units | Area ratio Mn/Fe | Area ratio average ± relative standard deviation (%) |
|---|---|---|---|---|
| | 1042 | 9505 | 0·110 | |
| 0·230 | 1055 | 9673 | 0·109 | 0·106(±4·2) |
| | 871 | 8651 | 0·101 | |
| | 936 | 9394 | 0·103 | |
| | 1826 | 8436 | 0·216 | |
| 0·410 | 2308 | 9622 | 0·240 | 0·224(±5·5) |
| | 1888 | 8874 | 0·213 | |
| | 2394 | 10566 | 0·227 | |
| | 2899 | 10147 | 0·286 | |
| 0·531 | 2271 | 8380 | 0·271 | 0·284(±3·4) |
| | 2729 | 9645 | 0·283 | |
| | 2141 | 7280 | 0·294 | |
| | 2703 | 9003 | 0·300 | |
| 0·570 | 2344 | 7934 | 0·295 | 0·302(±2·1) |
| | 2168 | 7153 | 0·303 | |
| | 2459 | 7922 | 0·328 | |
| | 2926 | 8824 | 0·332 | |
| 0·610 | 2668 | 8354 | 0·319 | 0·321(±4·0) |
| | 2422 | 8002 | 0·303 | |
| | 2937 | 8947 | 0·328 | |
| | 3486 | 7261 | 0·480 | |
| 0·918 | 3947 | 8381 | 0·471 | 0·482(±1·9) |
| | 4448 | 9015 | 0·493 | |
| | 4591 | 9512 | 0·483 | |

100 μm and 2 mm distance between sample road and the graphite counter electrode. The peak at 293·31 nm was selected, and the ratio of its integrated intensity around the maximum in relation to the Fe reference line (also integrated) at 292·66 nm was calculated. The iron emission line can be used as reference because its concentration in the steel samples varies only from 97·6 to 98·4%. Table 1 shows the values for the ratio of the integrated intensities and their relative standard deviation (RSD) estimated from four determinations of each sample. A linear calibration curve was obtained and the best fitting equation is

$$C_{Mn} = -0{\cdot}0069\ (\pm 0{\cdot}0096) + 0{\cdot}538(\pm 0{\cdot}016) \times R_{Mn/Fe}$$

with a correlation coefficient of 0·9982, where $R_{Mn/Fe}$ is the ratio of the integrated intensities and $C_{Mn}$ is the Mn concentration in the steel expressed in parts per cent (w/w). An average absolute standard deviation of 0·032% for the Mn concentration in the samples was estimated.

The technique, known as rotating disc, is frequently used to deliver liquid samples to the arc/spark excitation source [23–25]. In the evaluation of this technique with the automated spectrograph a d.c. spark excitation mode (125 μH, 15 μF, residual resistance) was employed. A 3 cm diameter graphite disc was spun with the standard accessory of the PGS2 and was dipped 1·5 mm into 400 μl solution containing Pb prepared in 0·1 mol/l hydrochloric acid with 1000 mg/l of K and placed in a porcelain cuvette. The graphite counter electrode was placed 3 mm away from the disc and a pre-burning and integration time intervals of 30 and 15 s respectively were employed. A cleaning procedure was adopted between measurements so that the same graphite disc could be used to perform up to 30 determinations. This procedure requires the removal of the sample, replacement by a 0·1 M HCl solution and the application of the excitation source for 45 s. The cleaning efficiency could be observed by exciting the disc without the sample solution and looking for the occurrence of the 405·78 nm lead atomic emission line. Usually, the cleaning procedure needs to be repeat twice.

The ratio between the integrated intensity under the emission line at 405·78 nm and the line under the potassium line centred at 404·41 nm was used to build a calibration curve that showed a linear behaviour in the range 3–50 mg/l of the metal described by the equation: $C_{Pb} = 0{\cdot}068(\pm 0{\cdot}009) + 0{\cdot}02034(\pm 0{\cdot}00033) \times R_{Pb/K}$, with a correlation coefficient of 0·9994, where $R_{Pb/K}$ is the ratio of the integrated intensities for Pb and K, and $C_{Pb}$ is the Pb concentration in mg/l. A mean relative standard deviation of the ratio of the integrated intensities of 4·1% was observed for the average of four replicates of each Pb solution containing 3, 5, 10, 20, 30, 40 and 50 mg/l of the metal.

### Conclusion

Photodiode arrays are now much more sensitive than early devices [6]. Sensor arrays can be used in spectrographs and this paper describes an approach for automating spectrographs at reasonable cost for qualitative and quantitative procedures. The automated instrument is simple to operate and may not require skilled operators.

The main disadvantage of the low-cost modernization proposed in this paper, when compared with other previously reported approaches [9, 10], is the long time that is required to scan a complete spectrum in order to carry out a qualitative determination. Typically, scanning the 26 windows would take about 10 min and some care is needed in periodically moving the sample to expose fresh portions of the sample for excitation, particularly if volatile elements are present. To minimize this problem, an expert system is being developed to find the minimum number of windows necessary to have enough qualitative information to identify the presence of 42 common elements. Preliminary results show that 15 windows are enough to observe at least two well characterized emission lines for the elements.

The automated spectrograph was found to be versatile in quantitative procedures. Concentrations of major components of steels could be found with reasonable precision and directly in the solid sample without pre-treatment, while concentrations as low as 3 mg/l of Pb could be determined in aqueous solutions.

Based on the results described and on the fact that few and simple modifications were necessary to the original spectrograph, it is concluded that photodiodes arrays despite other expensive image detectors (such as CID and CCD now available), are still capable of providing a very good qualitative and quantitative performance in regard to the modernization of old spectrographs.

### Acknowledgements

The authors wish to thank the FAPESP for research (92/1339-3) and fellowship (92/4298-6) grants and USIMINAS S.A. for providing the steel samples used in this work.

### References

1. Török, T., Mika, J. and Gegus, E., *Emission Spectrochemical Analysis* (Adam Hilger, Bristol, 1978).

2. Slavin, M., *Emission Spectrochemical Analysis* (Wiley-Interscience, New York, 1971).

3. Yudelevich, I. G. and Troshkova, G. P., *Analysis*, **20** (1992), 341.

4. Venkatasubramanian, R. and Karanjikar, N. P., *Analytical Letters*, **22** (1989), 981.

5. Danzer, K., Wagner, M. and Wienne, D., *Acta Chimica Hungarica*, **128** (1991), 623.

6. Codding, E. G. and Horlick, G., *Applied Spectroscopy*, **27** (1973), 366.

7. Codding, E. G. and Horlick, G., *Spectroscopy Letters*, **7** (1974), 33.

8. Horlick, G., Codding, E. G. and Leung, S. T., *Applied Spectroscopy*, **29** (1975), 48.

9. Brett, L., Stahl, R. G. and Timmins, K. J., *Analytical Spectrometry*, **4** (1989), 333.

10. Brett, L., Kalsi, A., Cummings, S. and Thombs, D., *Analytical Proceedings*, **28** (1991), 224.

11. Bye, C. A. and Scheeline, A., *Applied Spectroscopy*, **47** (1993), 2031.

12. Pomeroy, R. S., Jalkian, R. D. and Denton, M. B., *Applied Spectroscopy*, **45** (1991), 1120.

13. Pomeroy, R. S., Kolczynski, J. D. and Denton, M. B., *Applied Spectroscopy*, **45** (1991), 1111.

14. Wirz, D. F. and Blades, M. W., *Analytical Chemistry*, **58** (1986), 51.

15. Danzer, K. and Wagner, M., *Fresenius' Journal of Analytical Chemistry*, **346** (1993), 520.

16. Danzer, K., Venth, K. and Wagner, M., *Fresenius' Journal of Analytical Chemistry*, **350** (1994), 339.

17. Souza, P. S. and Pasquini, C., *Laboratory Microcomputer*, **9** (1990), 77.

18. Cunha, I . B. S. and Pasquini, C., *Analyst*, **117** (1992), 905.

19. Malcome-Lawes, D. J., *Laboratory Microcomputer*, **6** (1987), 16.

20. Malcome-Lawes, D. J., Pasquini, C. and Wong, K. H., *Laboratory Microcomputer*, **8** (1989), 44.

21. Malcome-Lawes, D. J., *Laboratory Microcomputer*, **6** (1987), 122.

22. EG&G Reticon, *Operational and Alignment Procedures for RC1000/1001*, California, USA (1992).

23. Kopp, J. F. and Kroner, R. C., *Applied Spectroscopy*, **19** (1965), 155.

24. Ondrick, C. W., Suhr, N. H. and Medlin, J. H., *Applied Spectroscopy*, **23** (1969), 111.

25. Kántor, T., Erdey, L. and Szabó-Ákos, Zs., *Talanta*, **17** (1970), 1199.

**Appendix 1.**

The QuickBasic 4.5 subprograms listed below assume that the parallel user port and the addressable interface have been properly initialized and that some variables have been defined in the main program [17, 21].

```
SUB zero (ad%, chsl%, chsh%, clsh%, clsl%)  'this subprogram resets the photodiodes
                                            'to null signals by charging the associated
                                            'capacitors

        CALL initarray (ad%, chsl%, chsh%, clsh%, clsl%)   'see comments below

FOR t = 1 to 2

        FOR n = 1 TO 1024

                FOR Q = 1 TO 30: NEXT

                mspeci!(n) = 0

                CALL outda(ad%, chsl%)  '0000 0010   'clock line high

                CALL outda(ad%, clsl%)  '0000 0000   'clock line low

        NEXT n

NEXT t

END SUB

SUB readarray (ad%, chsl%, chsh%, clsh%, clsl%)  'this subprogram reads the 1024
                                                 'values of the integrated intensities
                                                 'of the photodiodes in the array

        CALL initarray (ad%, chsl%, chsh%, clsh%, clsl%)  'see comments below

        FOR n = 1 TO 1024                    'performs the 1024 readings

                CALL readinter(251, di%)     'see comments below

                        dl% = di%

                CALL readinter(247, di%)

                        mspeci!(n) = (dl% * 16) + di%   '12 bits resolution data stored in

                                                        'mspeci!()

                        CALL outda(ad%, chsl%)

                        CALL outda(ad%, clsl%)          'see comments below

        NEXT

END SUB

SUB initarray (ad%, chsl%, chsh%, clsh%, clsl%)  'this subprogram initialize the
external
                                                 '"start" control line and leaves the
                                                 'external "clock" ready to the
                                                 'measurement of  the first photodiode
```

```

    CALL outda(ad%, 0)              'binary numbers sent to the output latch

        CALL outda(ad%, chsl%)      '0000 0010, clock high and start low

        CALL outda(ad%, clsl%)      '0000 0000, clock low and start low

    FOR Q = 1 TO 20: NEXT

        CALL outda(ad%, clsh%)      '0000 0001, clock low and start high

        CALL outda(ad%, chsh%)      '0000 0011, clock high and start low

        CALL outda(ad%, clsh%)      '0000 0001, clock low and start high

    FOR Q = 1 TO 20: NEXT

        CALL outda(ad%, clsl%)      '0000 0000, clock low and start low

        CALL outda(ad%, chsl%)      '0000 0010, clock high and start low

        CALL outda(ad%, clsl%)      '0000 0000, clock low and start low

END SUB

SUB readinter (ad%, di%)    'reads a integer of 8 bits from the interface. See references
                            '17 e 21 for details on the values of the variables OHSTR%
                            'OLSTR%, OACK%, IHSTR%, ILSTR% and IACK% that
                            'controls the communication handshaking
  OUT PB%, OHSTR%
   OUT PA%, ad%
    OUT PB%, OLSTR%
  WHILE (INP(PC%) AND OACK%) = 0: WEND
         OUT PB%, OHSTR%
          OUT PA%, 255
           OUT PB%, IHSTR%
            OUT PB%, ILSTR%
  WHILE (INP(PC%) AND IACK%) = 0: WEND
        di% = INP(PA%)        'di% contain the reading
      OUT PB%, IHSTR%

END SUB

SUB outda (ad%, bytetosend%)        'outputs an 8 bits integer (bytetosend%) to
                                    'address (ad%)
   OUT PB%, OHSTR%
    OUT PA%, ad%
     OUT PB%, OLSTR%
  WHILE (INP(PC%) AND OACK%) = 0: WEND
    OUT PB%, OHSTR%
     OUT PA%, bytetosend%
      OUT PB%, OLSTR%
  WHILE (INP(PC%) AND OACK%) = 0: WEND
    OUT PB%, OHSTR%

END SUB
```

